# Chromatic Illumination Discrimination Ability Reveals that Human Colour Constancy Is Optimised for Blue Daylight Illuminations

**DOI:** 10.1371/journal.pone.0087989

**Published:** 2014-02-19

**Authors:** Bradley Pearce, Stuart Crichton, Michal Mackiewicz, Graham D. Finlayson, Anya Hurlbert

**Affiliations:** 1 Institute of Neuroscience, Faculty of Medical Sciences, Newcastle University, England, United Kingdom; 2 School of Computing Sciences, University of East Anglia, Norwich, England, United Kingdom; University of Sussex, United Kingdom

## Abstract

The phenomenon of colour constancy in human visual perception keeps surface colours constant, despite changes in their reflected light due to changing illumination. Although colour constancy has evolved under a constrained subset of illuminations, it is unknown whether its underlying mechanisms, thought to involve multiple components from retina to cortex, are optimised for particular environmental variations. Here we demonstrate a new method for investigating colour constancy using illumination matching in real scenes which, unlike previous methods using surface matching and simulated scenes, allows testing of multiple, real illuminations. We use real scenes consisting of solid familiar or unfamiliar objects against uniform or variegated backgrounds and compare discrimination performance for typical illuminations from the daylight chromaticity locus (approximately blue-yellow) and atypical spectra from an orthogonal locus (approximately red-green, at correlated colour temperature 6700 K), all produced in real time by a 10-channel LED illuminator. We find that discrimination of illumination changes is poorer along the daylight locus than the atypical locus, and is poorest particularly for bluer illumination changes, demonstrating conversely that surface colour constancy is best for blue daylight illuminations. Illumination discrimination is also enhanced, and therefore colour constancy diminished, for uniform backgrounds, irrespective of the object type. These results are not explained by statistical properties of the scene signal changes at the retinal level. We conclude that high-level mechanisms of colour constancy are biased for the blue daylight illuminations and variegated backgrounds to which the human visual system has typically been exposed.

## Introduction

Evidence suggests that the human visual system is optimised for the environment in which it evolved, particularly at retinal and thalamic levels where spatial and spectral sensitivities have been shown to be tuned to natural scene statistics [1]–[4]. Although the ecological theory of perception would also suggest that higher cortical mechanisms are sculpted by natural scene statistics through evolutionary pressure [5], there is less direct evidence for such optimisation, particularly for mechanisms underlying colour perception. Colour constancy – the phenomenon by which object colours are perceived as constant despite changes in the illumination spectrum – is thought to involve mechanisms at the higher cortical level, in addition to retinal and thalamic components [6]–[9]. Here, we examine the hypothesis that *colour constancy* mechanisms per se are optimised for natural environments, and in particular, for natural illuminations.

The natural illuminations under which humans evolved are defined by the daylight locus, which describes the chromaticities of regular and typical variations of sunlight due to time of day, cloud-cover and geographical location, and closely parallels the chromaticities of black-body radiation at varying temperature, or the *Planckian locus* [10]. In industrial times, humans have also been exposed to manufactured light sources, including candlelight and incandescent lamps, and, most recently, fluorescent and solid-state light sources that have been designed to emulate neutral daylight illuminations [11]. When the illumination on a particular surface changes, the spectrum of light reflected from the surface also changes, although its intrinsic reflectance properties do not. In colour constancy, the human visual system has evolved mechanisms to keep surface colours constant across changes in the illumination, maintaining perception that closely corresponds to the unchanging surface reflectance properties rather than to variations in the reflected light [12].

Previous experiments investigating colour constancy have tested participants' ability to judge changes in colour appearance of uniform patches in scenes, under a small number of distinct illuminations [13]–[16]. These experiments have extensively probed mechanisms of colour constancy, but in general, with few exceptions [16], the experimental aims were not to elucidate under which illuminations these mechanisms perform best.

The surfaces used in colour constancy experiments are usually either simulated, using computer monitors, or are made from controlled paper with uniform chromaticities [17], with a few exceptions, in which real scenes have been shown under a small number of illuminations produced by a small number of fixed primary lamps [13], [18], [19], which are not representative of the natural range of global illuminations to which we are usually exposed. (In some of these experiments, additional spot lamps were used to illuminate a target surface only.) Where real scenes are used, these are also typically composed of generic, unfamiliar objects.

Although there is evidence that colour constancy improves as the number of surfaces within a scene increases [20]–[22], the notion that more complex characteristics of natural scenes may contribute to colour constancy – for example, via the memory colour of familiar objects providing a reference surface for colour calibration [12] – has not been adequately tested. Recent experimental evidence is divided, demonstrating heightened colour constancy for colour matches of Munsell papers in real scenes containing (among other fruits) a banana [19] but no effect of an image of a real banana image on colour matches of simulated patches [23]. These experiments address surface colour specifically, and while it is not clear whether colour constancy mechanisms are optimised for frequently encountered or natural surface colours, it is also unclear whether constancy mechanisms are biased towards illuminations to which we are commonly exposed.

Instead of matching colours of objects or surfaces under changing illuminations, here we introduce a new method of quantifying colour constancy using forced-choice illumination matching. In this method, observers first view a reference scene and then select from two successively presented scenes the one in which the illumination matches that of the reference scene. The surfaces and their spatial configuration are unchanged between the reference and alternatives; only the illumination changes. By systematically varying the illumination difference between the two alternatives, we obtain an illumination discrimination curve for each reference illumination. The rationale underlying this task as a measure of surface colour constancy is the same as that underlying asymmetric surface matching task measures [24], [25]. In the latter, observers typically adjust the chromaticity of a surface patch under a reference illumination to match its appearance under a test illumination. If the observer were perfectly colour constant, he would perceive as identical the two different chromaticities elicited by a fixed surface reflectance under two different illuminations. In practice, colour constancy is not perfect, and the matching chromaticity deviates from that predicted for a fixed surface reflectance. This deviation is typically cited as incomplete compensation for the change in illumination and therefore measures the lower limit of colour constancy under a fixed, typically large illumination change. Here, we instead measure the upper limit of colour constancy under varying illumination changes, by holding surface reflectances fixed and determining the range of illumination changes under which they indeed retain the same appearance. If an observer is unable to perceive a change in scene appearance under changes in illumination, then he is perfectly colour constant. If, conversely, the observer perceives a change in scene appearance and is therefore able to discriminate between illuminations, she is not perfectly colour constant.

It is important to note that, unlike in “operational colour constancy” studies [26], we are not measuring the ability of the observer to attribute a change in scene appearance correctly to a change in illumination versus a change in surface material, but instead measuring the ability of the observer to determine whether a change in scene appearance has occurred, explicitly under a change in illumination only. This method of illumination matching therefore probes colour constancy at the sensory level of appearance rather than a higher level of cognitive judgment. Because in the natural world, illuminations change more frequently than surface reflectances, this task provides a natural assessment of the limits of constancy: the limits of illumination change under which the visual system perceives no change in scene appearance.

**Figure 1:**
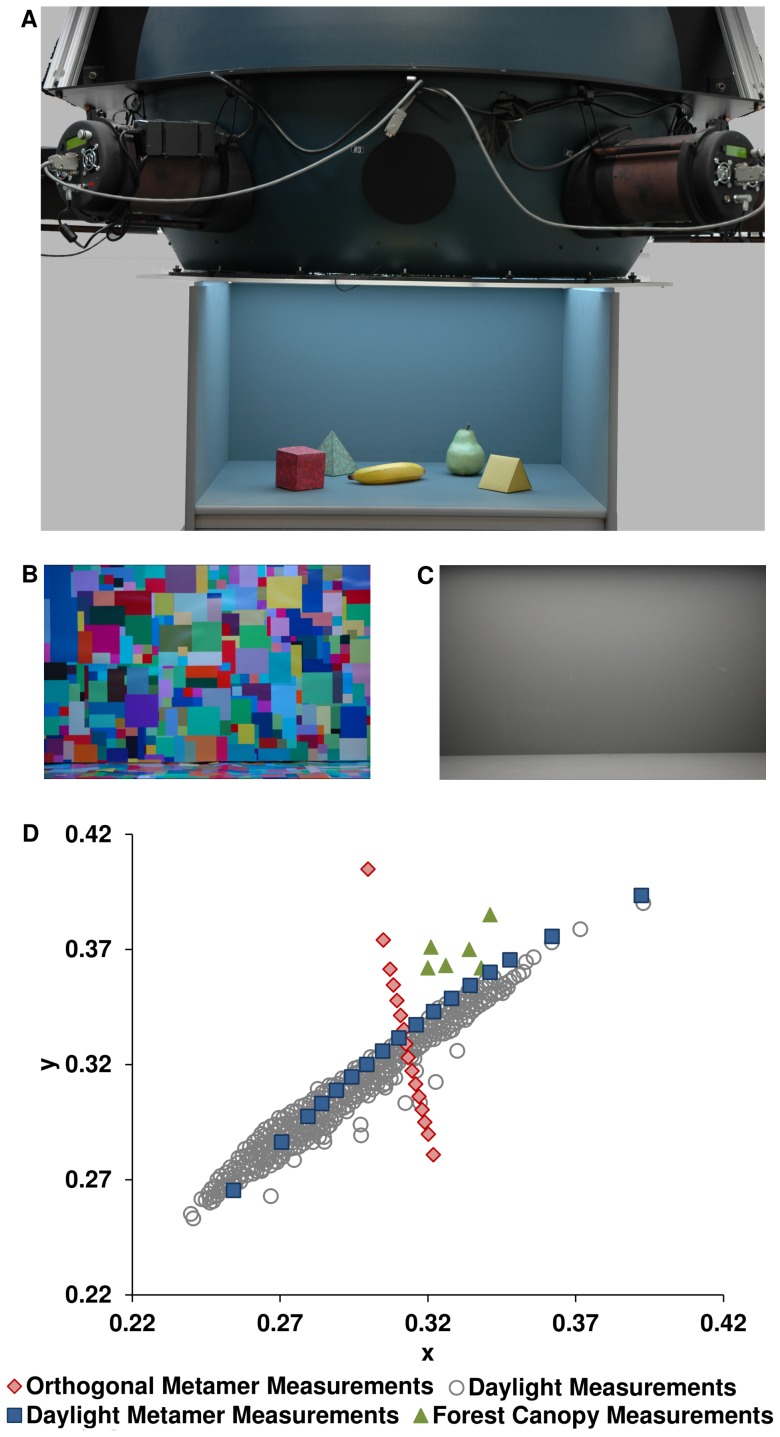
Photographs of the illuminator equipment and the scene backgrounds, with a plot of the chromaticity coordinates of illuminations used in the experiment. A. Photograph of illuminator and the viewing box (with front wall removed) under extreme blue illumination, with fake pear, banana and chromatically matched novel objects. B. The Mondrian background used for the variegated scene condition, under D67 illumination. C. The grey background used for the grey scene condition. D. Chromaticities of generated metamers atop daylight measurements taken and digitised from Hernandez-Andres et al. [36], in CIE 1931 colour space; green markers show chromaticities of Ugandan forest canopy illuminations measured by Sumner and Mollon [2].

We measure discrimination curves for systematically controlled changes in illumination, generated by a spectrally tuneable multi-channel LED light source, on real 3D surfaces (Figure 1A). The illuminations we use have broadband spectra and are either 1) metamers of daylight illuminations, or 2) atypical illuminations that share a correlated colour temperature with a central point on the daylight locus. Based on the premise that better illumination discrimination indicates poorer colour constancy, we test the following hypotheses: firstly, that illumination discrimination for atypical illuminations will be greater than for daylight illuminations; secondly, that illumination discrimination for scenes with a single uniform background surface will be greater than for those with multiple distinct surfaces; and third, that the presence of objects such as fruit, which have coevolved with human colour vision [3], will cue colour constancy mechanisms more effectively than chromatically matched, novel objects, and that therefore, illumination discrimination will be poorer for equivalent illumination changes on these scenes.

## Methods Overview

Participants were presented on each trial with a reference (target) illumination that illuminated a viewing box containing one of six scenes, with one of three scene content types (fruits – a real apple, banana, and a realistic fake pear; novel objects – three distinct 3D paper shapes with matched surface colours to the fruits; or no objects) and one of two backgrounds (uniform grey or Mondrian) (Figure 1B, 1C). Shortly after the target illumination had been presented, two test (comparison) illuminations were presented, successively, one of which was always identical to the target illumination, in a two-alternative forced choice task. Participants signalled on each trial which of the two comparison illuminations was the closest match to the target illumination. The target illumination was presented for 2000 ms and the comparisons each for 1000 ms with a 400 ms dark period separating each illumination. The difference between the target and comparison illuminations was systematically varied between trials to determine thresholds for illumination discrimination.

Illumination chromaticities varied along two distinct loci: the Commission Internationale de l'Éclairage (CIE) daylight locus and an orthogonal, atypical locus. The daylight locus closely parallels the Planckian (blackbody radiation) locus and varies from correlated colour temperatures of approximately 40000 K (blue-ish) to 4000K (yellow-ish) (Figure 1D). The atypical chromaticities were taken from the isotemperature line at 6700 K, which by definition is perpendicular to the Planckian locus in the uniform chromaticity plane at that point, computed according to the method established by Mori et al. (in Wyszecki and Stiles [10]). (Note that because of the way in which isotemperature lines are defined, they are of necessity not perpendicular to the Planckian locus when plotted in a non-uniform colour space, as in Figure 1D.) Chromaticities on this orthogonal curve varied along a roughly greenish-reddish (or cyan-magenta) axis. Two target chromaticities were selected on each locus, at ±10 perceptual steps (CIE ΔE_uv_ units) from D67 in the CIE Lu*v* colour space (see Figure 1D for a plot of all the generated chromaticties in CIE 1931 Yxy colour space, atop daylight measurements). For trials in which both comparison illuminations were the same as the target illumination (±0 ΔE_uv_ from target), one of the comparison intervals was arbitrarily pre-designated to be the correct choice, and therefore performance was expected to be at chance as the observer should be equally likely to pick either one of the two identical comparison illuminations. Performance data for these trials were indeed not significantly different from chance. Performance for comparison illuminations ±58 ΔE_uv_ from the target illumination, in which one comparison is identical to the target and the other an extreme change, did not differ from 100%. Therefore both of these trial types were removed from the statistical analysis; nonetheless, performance on these trials demonstrates that the task is meaningful and that observers comprehend its demands.

## Results and Discussion

### Illumination discrimination thresholds vary with chromatic direction and scene background

**Figure 2:**
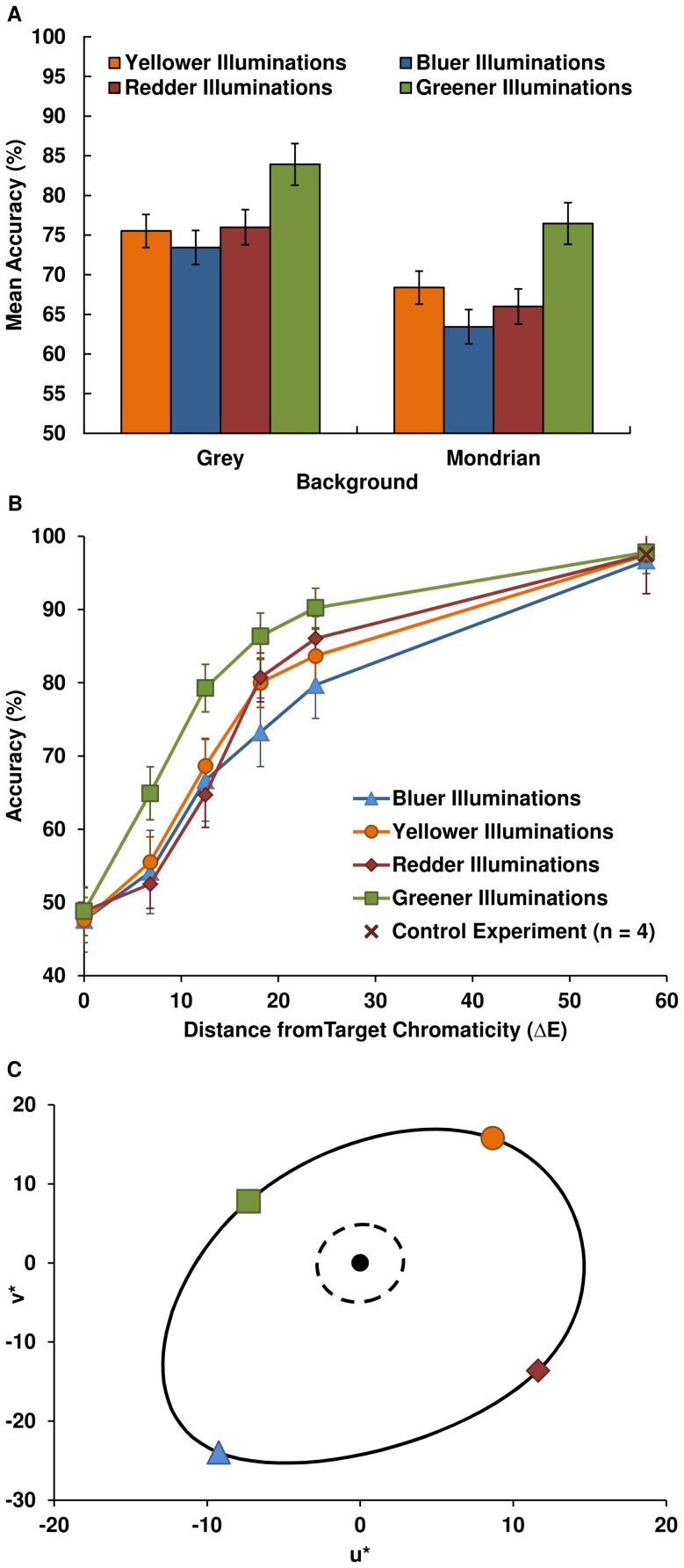
Mean discrimination accuracy for various conditions. A. Mean discrimination accuracy for illuminations by their chromatic direction, for conditions using the grey or Mondrian background; for significant differences see main text. B. Mean accuracy across all conditions and participants for each chromatic direction as a function of perceptual distance from the target chromaticity ΔE_uv_. C. Computed ΔE_uv_ mean thresholds at 75% accuracy for each chromatic direction, plotted in CIE u*v* colour space, with a spline forming the just-noticeable-difference discrimination contour (bold line) from D67 (black marker); just-noticeable-difference MacAdam ellipse boundary for D65 (dashed line) plotted around D67 point.

A repeated-measured ANOVA with three independent variables was used to analyse the data. The results demonstrate a significant performance difference between the daylight and orthogonal loci (*F*(1,7) = 17.404 p<.01), with mean discrimination accuracy (percent correct) lower for daylight illuminations (70.20% vs 74.74%), and mean accuracy across all illuminations and conditions equal to 72.47%. Mean discrimination accuracy for the grey backgrounds (μ = 76.37%) is significantly higher than for Mondrian backgrounds (μ = 68.57%; *F*(1,7) = 11.385, p = .012) (Figure 2A). No significant difference in discrimination accuracy is found for the different scene contents conditions: fruit, novel or no objects (*F*(2,6) = 1.776, p = .248).

For finer analysis of the illumination discrimination patterns, we divided each locus into two parts by splitting each locus at the center point (D67), thereby creating four loci of chromatic directions: bluer, redder, greener and yellower illuminations. A subsequent repeated-measures ANOVA with Greenhouse-Geisser corrections shows that over all conditions, mean accuracy differs significantly between chromatic directions (*F*(2.12,14.85) = 15.031, p<.01; Figure 2B). Illumination discrimination is poorest for bluer changes and most accurate for greener changes. Post-hoc tests using Tukey's HSD test shows performance on all chromatic directions to be significantly different between the Mondrian and grey background conditions (p<.05) with the exception of the greener illuminations; greener illuminations are, though, significantly different from bluer illuminations in each background condition separately (p<.01), and significantly different from the other chromatic directions (p<.05), while redder and yellower illuminations are not significantly different from each other but are from both bluer and greener illuminations (p<.05).

### Scene Statistics do not Predict Illumination Discrimination Asymmetries

Certain computational theories of colour constancy [6], [8] assume either that the scene surface reflectances average to neutral or that the brightest surface is white, thereby enabling an estimate of the illumination chromaticity to be gained from scene statistics. If scene statistics are the sole contributors to constancy mechanisms, we may expect their variation to explain the variation in performance under different illuminations that we observe here. For example, if the brightest-is-white strategy governs illumination estimation, we would expect discrimination performance to be greatest for those illumination changes in which there is greatest change in the visual signal from the brightest surfaces in the scene. We therefore examined in further detail the distributions of illumination change signals conveyed by the reflected light from surfaces across the entire box. To do so, we first took hyperspectral images of the grey and Mondrian box backgrounds under each of the 34 unique test illuminations (sampling the spectra at 4 nm intervals at each pixel in an image array of 1917×800 pixels), then selected 95 distinct patches at random in the Mondrian background image and analysed the spectra from these locations and from the exactly corresponding spatial locations in the grey background images. Spectra within each patch were averaged and converted to cone excitations. For each patch and each test illumination, the change in cone excitation elicited under the test illumination relative to the target illumination was computed in each of the three channels of luminance (L+M), red-green (L−M), and blue-yellow (S − [L+M]) in the modified MacLeod-Boynton (McB) cone-opponent contrast space [27]–[29] (see the description of cone contrast calculation in the Methods).

**Figure 3:**
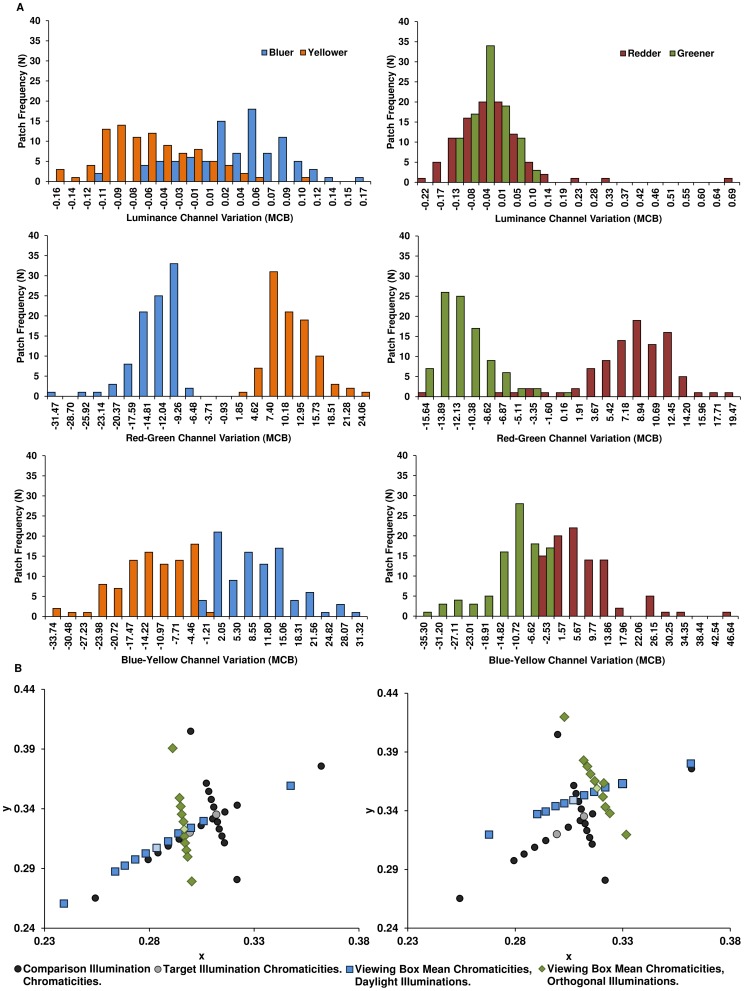
Histograms of changes in cone-opponent channel excitations of 95 surfaces between D67 and the bluer, redder, greener and yellower illuminations ±18ΔE_uv_ away in the Mondrian background condition, in modified MacLeod-Boynton (McB) coordinates. B. Mean scene chromaticities under each comparison illumination for one target illumination (grey symbol), for the daylight (blue symbols) and orthogonal (green symbols) loci, in CIE 1931 xy chromaticity coordinates. Illumination chromaticities are also shown (black symbols). Left: grey background condition. Right: Mondrian condition. Note that targets (lighter markers) are asymmetrically placed with respect to the crossing position.

Histograms of the McB channel changes are illustrated in Figure 3A, for the test illuminations at distance 18 ΔE_uv_ from the target illumination in each of the four chromatic directions, for the Mondrian background (for the grey background see Figure S1, in supplementary information). There is no visible cue from the shape or magnitudes of the change signal that would explain the asymmetries in performance between the chromatic directions; in particular, neither the maximum nor mean signal in any of the three channels is greater for the greener illumination change than for the other illumination change directions. (See Figure S2B, which explicitly compares maximum and mean changes, as well as skewness and kurtosis of the change distributions, for each illumination direction across all change increments, in the luminance channel). Statistical analysis confirms that there is no significant correlation between any of these characteristics (in any McB channel) and discrimination performance for that chromatic direction alone. For example, maximum luminance change for yellower illuminations does not correlate with mean performance for those illumination changes, but does correlate highly with performance for greener illuminations (r = .884, p<.05); moreover, the maximum luminance change for bluer illuminations correlates with performance on all but redder illuminations (r = .979, .960, .977; p<.05, for yellow, blue and green illumination changes respectively). Therefore, neither the maximum nor mean McB changes account for performance in any specific chromatic direction, or explain the observed chromatic biases, and therefore neither does the brightest-is-white assumption Furthermore, the possibility that observers are adopting the strategy of monitoring signal changes in a single Mondrian patch assumed to be white or neutral is excluded because (a) the Mondrian pattern deliberately contains no patches of neutral reflectance; (b) the pattern of McB changes between patches across illumination directions is highly variable, so that the observer would be unable to predict the identity of the brightest patch from trial to trial; and (c) the asymmetry in performance holds for the grey background, effectively a single patch, and is also unexplained by its distribution of McB channel changes.

Changes in the average scene chromaticity also do not explain performance differences between the Mondrian and grey backgrounds. Neither condition satisfies the grey world assumption [6], [30], [31]: the average scene chromaticity is not an accurate predictor of the scene illumination chromaticity for either background. The means of the 95 surface chromaticities are shown in Figure 3B. The scene average chromaticities are shifted relative to the illumination chromaticities, although the ΔE_uv_ intervals and relative positions of the test illuminations are roughly preserved. For both backgrounds, this shift is explained by the average surface reflectance not being perfectly neutral; in particular, the grey paper reflectance is slightly higher in the short-wavelength region compared to the long (as in figure S2A). As the number of surfaces in the scene increases, the distribution of mean chromaticities tightens, but not sufficiently to explain the difference in performance between Mondrian and grey backgrounds: for equivalent changes in mean scene chromaticity in the two backgrounds, performance is still significantly greater for the grey background (F(1,29) = 51.692, p<.001; repeated-measures ANOVA calculated from the interpolated performance curves as a function of box mean chromaticity under each comparison illumination). Moreover, performance (again as a function of box mean scene chromaticity) depends significantly on illumination direction (F(1.51, 43.86) = 77.318, p<.001), with post-hoc t-tests (performed as paired sample t-tests in which the interpolated performance on each illumination direction for the Mondrian condition was paired with the performance of that illumination direction for the grey condition) confirming that yellow, red and green illuminations are significantly different across background conditions (t(29) = 4.70, t(29) = 9.46, t(29) = 8.58, p<.05, respectively). The difference in performance between the Mondrian and grey backgrounds is therefore more likely to be due to the surface variegation itself. Moreover, the bias against illumination discrimination in the bluer direction and towards illumination discrimination in the greener direction is not explained by systematic differences in the mean scene chromaticity changes along the bluer or greener directions (see Figure 3B).

### Surface Chromatic Discrimination Thresholds do not Predict Illumination Discrimination Asymmetries

Asymmetries in chromatic discrimination for colour patch matching tasks are well described by discrimination ellipses [10], [32], [33], which illustrate just noticeable differences from a central chromaticity at each point along the ellipse contour, with chromaticities falling inside of the ellipse indistinguishable from the centroid chromaticities. It is natural to ask whether these surface chromaticity discrimination asymmetries explain the asymmetries in global illumination discrimination. We therefore compare the standard MacAdam ellipse [10] in this region of the chromaticity diagram with the approximate ellipse computed from the mean discrimination thresholds of observers for this task, in Figure 2C, (threshold values in ΔE_uv_ units: green 10.7; red 17.9; blue 25.7; yellow: 18.0). (Note that in the perceptually uniform CIE Lu*v* space the MacAdam ellipse becomes roughly circular. Also, because there is no standard ellipse centred on the D67 chromaticity, we have re-centred the D65 ellipse on the D67 chromaticity (u*v* = −4.905, 7.061), which sits at the just-noticeable-difference border of the D65 ellipse). The discrimination thresholds for the illumination discrimination task are much larger than the MacAdam ellipse, and asymmetric between the axes as well as between the unipolar directions of each axis. The general magnitude difference between the illumination judgment thresholds and the MacAdam ellipse is at least partly explained by the task differences: in this task, comparison illuminations are presented successively rather than simultaneously as is the case for patches in colour field matching experiments (e.g.Krauskopf and Gegenfurtner [32]), and the discrimination is global rather than local. While empirical results and models of chromatic discrimination of chromatically variegated surfaces [34], [35] suggest that elongation of discrimination ellipses (reduced sensitivity) occurs along directions of maximal chromatic variation within stimuli, the reduced sensitivity along the blue-yellow axis in this task cannot be explained by a bias in chromatic variation of the background or scene surfaces, as these vary significantly between the Mondrian and grey backgrounds (see Figure S3 in the supplemental information for the principal axes of chromatic variation), and the performance bias across illuminations is the same for both.

## General Discussion

In paradigms that use surface colour matching across illuminations to measure colour constancy, close matches to a target patch's surface spectral reflectance function require the visual system to discount the scene illumination; in the case of achromatic adjustment tasks, a perfect match would result in the patch appearing white while having the same chromaticity as the scene illumination [17]. We therefore propose that colour constancy may be measured using an illumination discrimination task for fixed surface reflectances, with poor discrimination of changes in scene illumination signalling conservation of scene appearance and therefore good colour constancy and, conversely, high change discrimination signalling poor constancy. That is, if the observer is unable to perceive a change in surface colour appearance under changes in illumination, he is perfectly colour constant. Illumination discrimination was poorest for bluer illuminations along the daylight locus, indicating heightened colour constancy for these illuminations over all others. Poorest colour constancy is experienced in the greener illuminations along the orthogonal locus, for which discrimination between illuminations was best.

The results demonstrate clear differences between chromatic directions, with the least typical illuminations eliciting the best discrimination. Bluish illuminations are the most common among daylight illuminations, followed by yellowish illuminations, then by the rarer reddish illuminations experienced near sunset [36], and lastly by greenish illuminations, experienced only in scenes with dense forestation [28], [37], and displaced from the daylight locus as demonstrated by measurements from the Ugandan forest canopy [2] (see Figure 1D). The accuracy of illumination discrimination follows this pattern, with illumination changes that are more common in nature discriminated less effectively. The asymmetry within and between axes suggests a bias that is not seen in surface colour discrimination. Other studies of colour constancy have reported chromatic direction biases; for example, better colour constancy is reported for illumination shifts in the blue-yellow direction compared to shifts in the red-green direction [25] , in an asymmetric surface matching task, partly explained by a cone-opponent adaptation model, but demonstrated only for a small number of fixed shifts in unnatural illuminations (mixed narrow-band) and without systematic exploration of chromatic axes. Accelerated chromatic adaptation to greenish shifts in surface colours of heterochromatic stimuli at very short time scales, as measured by the shift in corresponding achromatic point [38], has also been reported. These results suggest differences in the dynamics of chromatic adaptation between chromatic directions and are generally consistent with ours in demonstrating improved performance in the greenish direction, but imply the opposite consequence for colour constancy. The difference in methodology between these studies and ours precludes further detailed comparison. Moreover, although surface discrimination studies also find evidence for higher blue-yellow thresholds (an elongated blue-yellow axis), and enhanced discrimination along the red-green axis [32], [39], as shown in our data, this is the first evidence for enhanced change discrimination specifically in the green direction and not mirrored in the red direction.

The results also indicate significantly poorer illumination discrimination, and therefore stronger colour constancy, for the variegated (Mondrian) background relative to the uniform grey background, across all chromatic directions. Previous studies using self-luminous computer displays have demonstrated that as the number of surfaces with distinct chromaticities presented to the viewer increases – in other words, as the scene articulation increases – so does the viewer's ability to attribute changes in surface chromaticity correctly to a (simulated) illumination or surface reflectance change [22], [38], [40]. In the current task, the viewer is required to make all judgments solely on the basis of an illumination change; that is, the observer is informed explicitly that only the illumination will change, and is aware that the configuration and surface reflectances of patches in the Mondrian scene do not vary across illuminations. The difference in performance between the grey and Mondrian backgrounds suggests that as the scene articulation increases, colour constancy improves because illumination changes become less discriminable, not because more information about the illumination *per se* is available from the greater number of surfaces. The differences in accuracy per chromatic direction are nonetheless preserved in both conditions, which suggests a universal bias that is preserved across scene contents. Crucially, this bias, perhaps affected by levels of articulation in a scene, is independent of the surface qualities within a scene.

Contrary to the hypothesis that the presence of real, familiar objects will drive colour constancy mechanisms more effectively than chromatically matched, novel objects, we found no significant difference in performance for scenes containing fruits in comparison to novel objects, across all illumination and background conditions. This lack of a familiarity effect might be due to the information articulated by the local surround outweighing that from the fruits or novel objects, for both the grey and Mondrian backgrounds. We suggest that silencing the background signal or focussing attention on the object itself may be necessary to reveal an effect of object familiarity, and are therefore examining this possibility in further experiments.

Contrary to certain computational models of colour constancy [6], [8], low-level image statistics do not explain the illumination discrimination performance, as demonstrated by analysis of the signals available to the initial cone-opponent contrast encoding pathways, obtained from hyperspectral images of the entire scene under the varying illuminations. In particular, there is a significant performance bias for blue illumination, with no corresponding bias in the statistics-based signals. It is therefore difficult to explain the performance differences between chromatic directions in terms of statistics-based signal processing at early levels in the visual pathway. The results instead lend weight to the notion that higher-level cortical mechanisms contribute significantly to colour constancy and that these are optimised for the natural environment. This conclusion is broadly consistent with other reports of optimisation and bias at higher levels in the visual pathway. For example, dichoptic presentation of scenes has been shown to affect levels of chromatic adaptation, placing at least some of the underlying mechanisms in the cortex [38]. Early cortical organisation of colour and orientation processing has also been shown to reflect the statistical properties of natural images [5]. It is also consistent with the notion that the visual system has been shaped by colours in natural scenes to which we have been exposed. The primary axis of variation in colour signals from natural images falls along the blue-yellow axis in modified McB space, for earth and sky images [29], [41]. This variation is, in turn, likely to arise largely from variations in natural illumination along the blue-yellow daylight locus [29]. The visual system may therefore benefit from silencing responses to typical blue-yellow variations in favour of heightened discrimination for atypical changes along the red-green axis, which are more likely to correspond to changes in objects rather than illuminations. In embedding this bias towards illumination chromaticities (blue rather than green) to which it has typically been exposed during human evolution, the visual system thus gains the ability to distinguish between meaningful and non-meaningful variations in the environment.

## Methods

### Ethics Statement

The experiment was conducted in accordance with the APA Ethical Principles, and was granted ethical approval by the Ethics Committee of the Faculty of Medical Sciences at Newcastle University (reference number 00312). Participants were asked to give written consent before participating in the study, and were informed of their right to withdraw at any time, without penalty.

### Participants

Eight observers (6 female; mean age 26 y; range 20–28) participated in the study, all naïve to its purposes. All participants were recruited by opportunity sampling through the Institute of Neuroscience Research Volunteer Program on a first-come, first-serve basis. All participants had normal or corrected to normal visual acuity, and no colour vision deficiencies, as confirmed by testing with the Ishihara Colour Plates and the Farnsworth-Munsell 100-Hue Test (mean total error score 25 [42]). Participants were paid £7 per hour for their participation in the study, at the end of each experiment session.

### Design

A two-alternative forced-choice task was used in a 2×2×3 repeated-measures design. The independent variables were the illumination sets (locus type: daylight or atypical) that illuminated the viewing box, and the contents of the viewing box, which was lined with either Mondrian or grey card, and contained either no objects, fruits or novel objects.

### Apparatus

A spectrally tuneable illuminator was used, consisting of 6 LED (Gamma Scientific RS5B) light sources, each with a bank of 10 programmable LED channels, which project into an integrating sphere, which in turn emits the combined light into a viewing box, producing diffuse, nearly uniform illumination onto the contents of the box [43]; see Figure 1A. The viewing box was 71 cm (width)×77 cm (depth)×47 cm (height), with a viewing aperture of 7.5 cm height and 14.5 cm width built into the front wall of the box, situated centrally 9.5 cm from the top of the box. A gaming pad was linked to a computer running Windows 7, MATLAB 2011b and custom software, which also controlled the illuminator. The computer was equipped with an ASIO enabled sound card, to provide low-latency audio, which was outputted to headphones.

### Stimuli

The viewing sides, back wall, and floor of the viewing box were lined with either standard uniform matte grey poster board (with mean CIE 1931 coordinates x = 0.299, y = 0.324, under the D67 illumination), or Mondrian paper (x = 0.321,y = 0.359, under D67; see Figure 1, C & B respectively), and contained either no objects, fruits (an apple, banana and realistic fake pear), or novel 3D primitives constructed from paper card (see Figure 1A for example). The Mondrian paper was inkjet-printed on non-glossy paper. The Mondrian patches varied in size from 0.2 cm–12.0 cm, or roughly 7.6 degrees of visual angle for the largest patch size at the viewing distance of 90 cm. The paper surfaces of the primitives were printed with an all-over multi-coloured random squares pattern, in which the individual square colours were colorimetrically matched to the real fruit surface colours under D67 illumination (a cube matched with an apple, a triangular prism with a banana and a pyramid with the pear), using a calibrated ink-jet printer (see Table S2 for tabulated chromaticities). Hyperspectral reflectance data of the background surfaces are available from the corresponding author on request.

Two sets of illuminations – 17 samples each from the daylight locus and an orthogonal locus – were created (see following section). The chromaticities of the 2 target illuminations on each locus were ±10 perceptual steps (ΔE_uv_ units) from D67 in the CIE Lu*v* colour space (see Figure 3B). The chromaticities of the 11 comparison illuminations were ±0, 6, 12, 18, 24 or 58 ΔE_uv_ from each target, as described in the main text.

### Illumination Generation, Measurement and Calibration

To generate the illuminations, a set of chromaticities for the target and test illuminations, separated by the desired ΔE_uv_ intervals (as above), were selected from the two loci. The spectral power distribution of each type of LED at 11 different intensities (1% and 10–100% in steps of 10) was measured inside the illuminator's integrating sphere using a PR650 spectroradiometer. These readings were used to produce a set of calibrated basis functions, which were in turn used to calculate the closest achievable matching illumination using the colorimetric match method we have previously described [43]. This method compensates for the intensity-dependent peak-wavelength shift exhibited by each LED channel in the Gamma Scientific RS-5B lamps, and seeks illumination spectra whose shape matches the desired spectrum shape in the least-squares error sense and whose CIE chromaticity coordinates precisely match the chromaticity of the desired spectrum. This method is possible for the daylight locus for which standardised spectra exist, but not for the orthogonal locus. We therefore imposed an additional constraint of maximal smoothness for the matching spectra on both loci. The final constraint imposed was for constant overall luminous flux across all illuminations.

To implement these constraints, we adapted the metamer sets approach from Finlayson and Morovic [44]. Metamer sets were computed for each desired chromaticity using linear models for the LED channels at each of several intensity ranges. To select the smoothest metamer for each chromaticity, quadratic programming was used to find the convex combination of the spectra at vertices of the metamer set convex hull whose smoothness is maximal.

The resulting spectra for the most extreme chromaticity changes are shown in Figure S2. The constant luminous flux constraint was well met: the measured luminance of a fixed position in the white integrating sphere varied less than 0.46% around a mean of 78.34 cd/m2 across all 34 illuminations. The luminance of a white calibration tile inside the viewing box varied between 22.49 and 23.85 cd/m2 across all 34 illuminations (see Table S1 for tabulated chromaticities). Repeated spectroradiometric measurements of the LED channel basis functions and the 34 test illuminations taken during and after the experimental sessions ensured that the desired spectra were maintained; measurements of the full metamer set showed a mean change of 1.19 ΔE_uv_ over the 6 weeks of testing.

### General procedure

Participants were seated in front of the viewing box and asked to look through the viewing aperture. Their heads were not fixed, but their viewing distance from the scene was constrained by the box front, which contained the viewing hole. The scene was not initially visible as the box was not illuminated. Participants were given standardised instructions for the experiment, and were directed towards two marked buttons on the gaming pad that signalled either 1 or 2. Participants were asked to use these to indicate which of 2 lights shown was the closest match to the initial light shown in each trial. The instructions read: “*You will be shown a light that illuminates the viewing box; this is the target light. Then there will be two subsequent lights, you are asked to signal which is most like the target light, using either of the buttons,* [*1*] *denoting the first light is most similar, or* [*2*] *for the second light*”. A 2-minute dark adaptation period preceded the start of the main experiment.

Each trial began with three audible tones delivered through the participant's headphones, signalling the start of a new trial. The box was immediately illuminated by the selected target illumination, which remained on for 2000 ms. The illumination was switched off and the box remained dark for 400 ms, before another tone signalled the first comparison illumination which illuminated the viewing box for 1000 ms. The box then went dark for a further 400 ms before another tone signalled the second illumination which illuminated the viewing box for 1000 ms. One of these two comparison illuminations was identical to the target illumination in every trial; the other comparison illumination was selected at random from a lookup table containing the 12 comparison illuminations for that target illumination (the 0 ΔE_uv_ comparison illumination was used twice, once for each of the two ± ΔE_uv_ sets), resulting in each comparison illumination being presented 10 times with the exception of each target illumination which was presented at least twice in each trial and 20 times as a comparison. The illumination presentation was time-locked to the sound presentation, with a delay measured at less than 30 ms.

The box then remained dark and a final tone cued participants to respond either 1 or 2 via the keypad. There was a minimum gap of 1000 ms between trials which factored in the time taken for participants to respond to the previous trial; trials were self-paced. Each participant completed 480 trials per condition (2880 total, 240 per locus, with two targets per locus, and 10 per comparison). Participants were given a mandatory 1 minute break after every 120 trials, but were also informed that they could break voluntarily at any time and return, or withdraw.

Each experimental condition was conducted in a separate session. Sessions were conducted at each participant's convenience and testing spanned a 6 weeks period.

### Control experiment

One of the comparison illuminations for one of the targets on one locus (the most extreme “red” comparison illumination, at +58 dE) (10 trials per participant) was not shown correctly; instead of the +58dE test illumination, the 0dE target was shown, due to a miscommunication between the controlling computer and the illuminator. Participants therefore performed not significantly different from chance on these trials, as the comparison illuminations were the same as the target. These 10 trials were treated as ±0 ΔE from target trials and were removed from analysis. To confirm the level of performance expected for this illumination, the communication fault was corrected and a control experiment conducted with 4 participants in which they performed the task as before, with the correct comparison illuminations, and using only the grey background condition with no objects present (all illuminations were tested, not just that which was not shown correctly). Accuracy for the extreme red comparison illumination in this control experiment was not significantly different from the ±58 ΔE comparisons at the other extremes, and not significantly different from 100%.

### Cone contrast calculation

The modified MacLeod-Boynton (McB) coordinates of each stimulus patch were computed as the scaled McB coordinates of the stimulus relative (in contrast) to the McB coordinates of the target illumination whitepoint. The McB coordinates *r_McB_* , *b_McB_*, and *lum_McB_* are defined as *l/(l+m)*, *s/(l+m)* and *(l+m)* respectively, where *l*, *m* and *s* are the long-, middle- and short-wavelength cone excitations of the stimulus. The contrast of the stimulus with respect to the target whitepoint is calculated by the following formulae (using the scaling factors of McDermott and Webster [29]):

Red-green contrast: 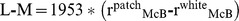


Blue-yellow contrast: 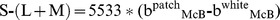


Luminance contrast: 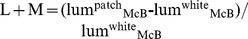


The change in McB coordinates for each patch under the test illumination relative to the target illumination was calculated by subtracting the McB coordinate values under the target illumination from those under the test illumination (note that the whitepoint for both sets of coordinates was held at that of the target illumination).

## Supporting Information

Figure S1 **Histograms of changes in cone-opponent channel excitations of 95 distinct background locations between D67 and the bluer, redder, greener and yellower illuminations ±18ΔE_uv_ away in the grey background condition, in modified MacLeod-Boynton (McB) coordinates.**
(TIFF)

Figure S2 **Scene statistics from the grey background condition.** A. top: Surface reflectance function of the grey background material (in blue) with .05 line marked (dashed line); below: Plots of relative spectral power for each of the four extreme metamer spectra: bluer, yellower, greener and redder, respectively. B. Maximum, mean, skewness and kurtosis values for cone-opponent contrast channel changes between D67 and the bluer, redder, greener and yellower illuminations at each ΔE_uv_ comparison in the grey background condition, in modified MacLeod-Boynton (McB) coordinates.
(TIFF)

Figure S3 **Chromaticity co-ordinates of 95 patches from the Mondrian background condition (top) and grey background condition (bottom) under D67 illumination.** The first principal components are marked with solid lines (slopes of 0.93 and −1.08, respectively); blue lines indicate the blue-yellow variation direction, and green lines the red-green variation direction, respectively. The greatest variance occurs along the blue-yellow direction in the Mondrian background, and along the red-green direction for the grey background.
(TIFF)

Table S1 **CIE 1931 xy chromaticity coordinates of readings of the 34 illuminations used in the experiment, for the two loci.**
(DOCX)

Table S2 **CIE 1931 xy chromaticity coordinates of the fruit and matched printed papers used in the experiment, measurements taken under D67.**
(DOCX)
