# Supplementary figures and images for: Chromatic Illumination Discrimination Ability Reveals that Human Colour Constancy Is Optimised for Blue Daylight Illuminations
Source: PLoS One. 2014 Feb 19;9(2):e87989. doi: 10.1371/journal.pone.0087989 (PMC3929610; doi:10.1371/journal.pone.0087989)

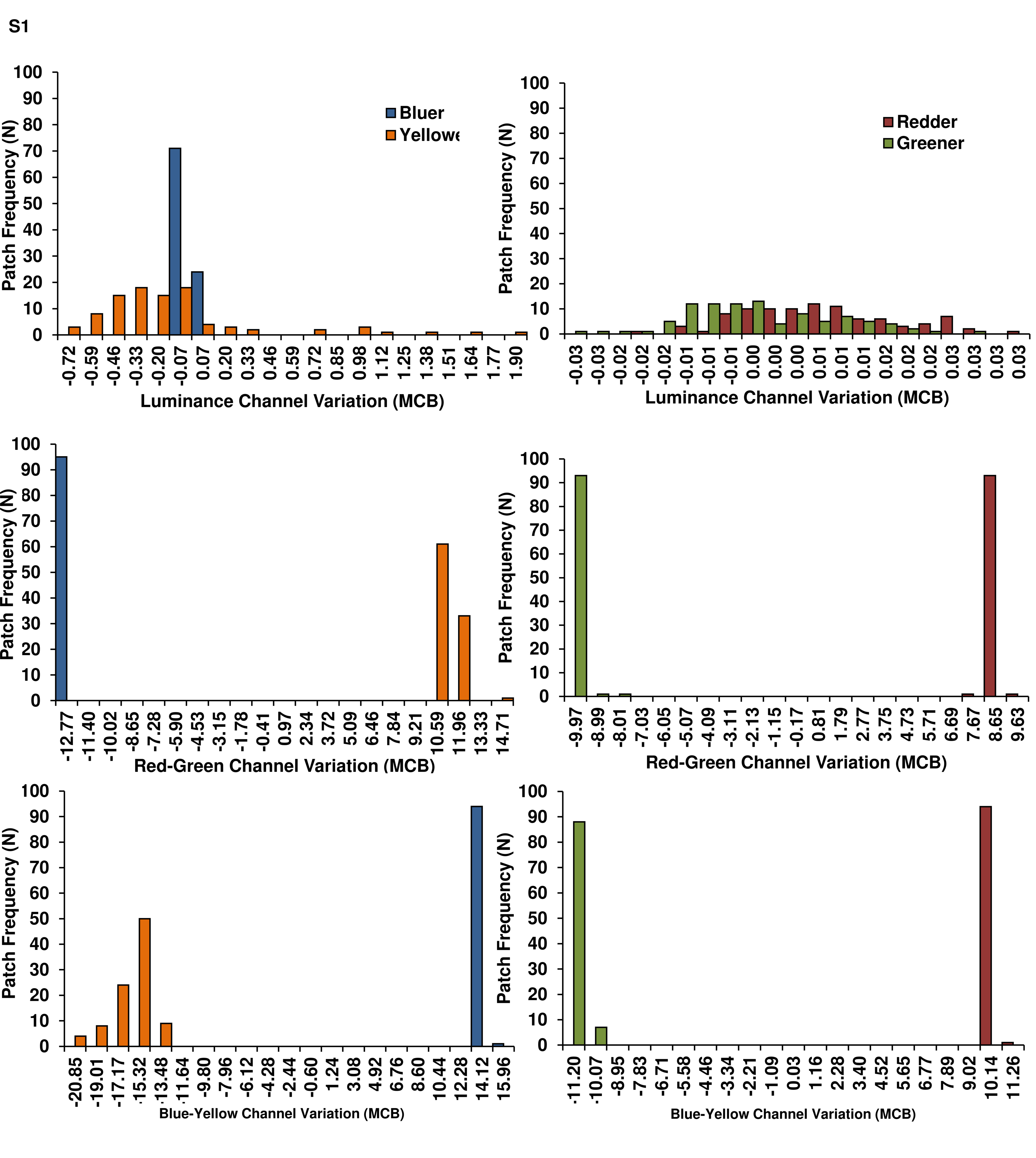

Supplement: Figure S1 — Histograms of changes in cone-opponent channel excitations of 95 distinct background locations between D67 and the bluer, redder, greener and yellower illuminations ±18ΔEuv away in the grey background condition, in modified MacLeod-Boynton (McB) coordinates. (TIFF) [file pone.0087989.s001.tif]

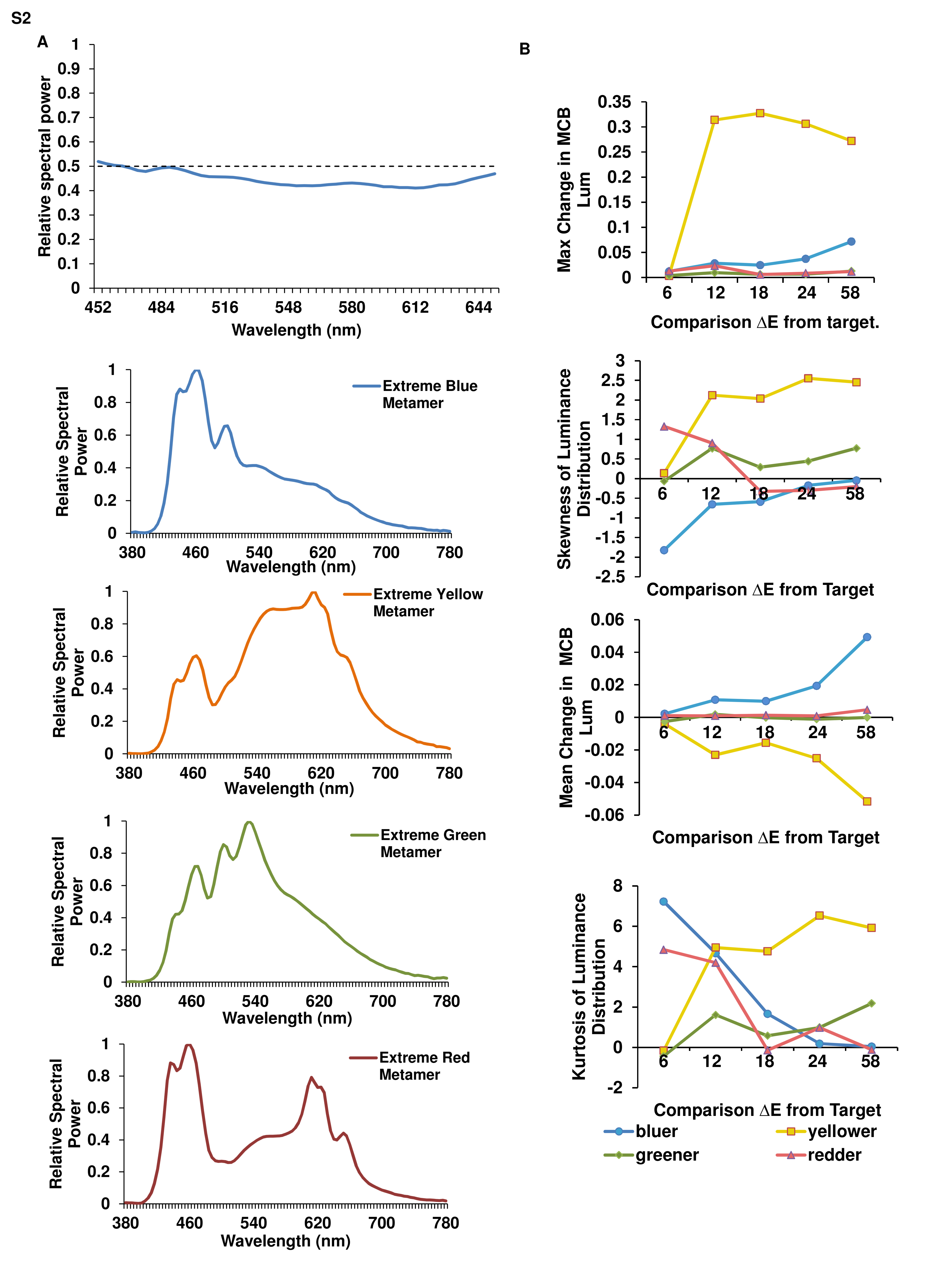

Supplement: Figure S2 — Scene statistics from the grey background condition. A. top: Surface reflectance function of the grey background material (in blue) with .05 line marked (dashed line); below: Plots of relative spectral power for each of the four extreme metamer spectra: bluer, yellower, greener and redder, respectively. B. Maximum, mean, skewness and kurtosis values for cone-opponent contrast channel changes between D67 and the bluer, redder, greener and yellower illuminations at each ΔEuv comparison in the grey background condition, in modified MacLeod-Boynton (McB) coordinates. (TIFF) [file pone.0087989.s002.tif]

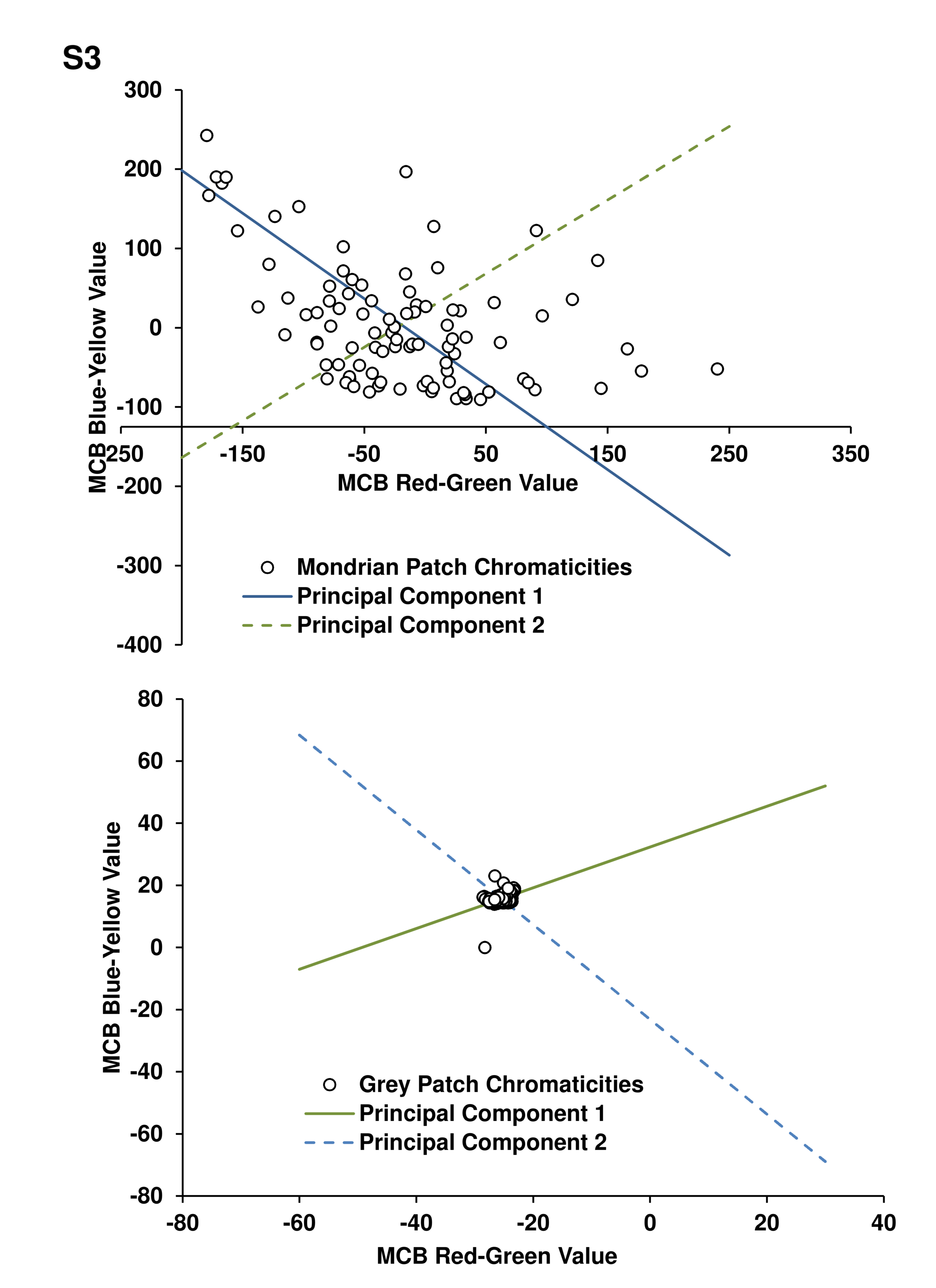

Supplement: Figure S3 — Chromaticity co-ordinates of 95 patches from the Mondrian background condition (top) and grey background condition (bottom) under D67 illumination. The first principal components are marked with solid lines (slopes of 0.93 and −1.08, respectively); blue lines indicate the blue-yellow variation direction, and green lines the red-green variation direction, respectively. The greatest variance occurs along the blue-yellow direction in the Mondrian background, and along the red-green direction for the grey background. (TIFF) [file pone.0087989.s003.tif]
