# Supplementary material for: Chromatic Illumination Discrimination Ability Reveals that Human Colour Constancy Is Optimised for Blue Daylight Illuminations
Source: PLoS One. 2014 Feb 19;9(2):e87989. doi: 10.1371/journal.pone.0087989 (PMC3929610; doi:10.1371/journal.pone.0087989)
Supplement: Table S1 — CIE 1931 xy chromaticity coordinates of readings of the 34 illuminations used in the experiment, for the two loci. (DOCX) [file pone.0087989.s004.docx]

Table S4. CIE 1931 xy chromaticity coordinates of readings of the 34 illuminations used in the experiment, for the two loci.

| **Daylight Locus** |  | **Orthogonal Locus** |  |
| --- | --- | --- | --- |
| **x** | **y** | **x** | **y** |
| 0.2993 | 0.3201 | 0.3119 | 0.3352 |
| 0.3219 | 0.343 | 0.3158 | 0.3115 |
| 0.2542 | 0.2653 | 0.2997 | 0.4049 |
| 0.2705 | 0.2864 | 0.3049 | 0.3741 |
| 0.2794 | 0.2975 | 0.3072 | 0.3614 |
| 0.2841 | 0.3031 | 0.3083 | 0.3546 |
| 0.289 | 0.3088 | 0.3095 | 0.3478 |
| 0.2942 | 0.3146 | 0.3107 | 0.3414 |
| 0.3047 | 0.3259 | 0.3125 | 0.329 |
| 0.3102 | 0.3316 | 0.3133 | 0.3231 |
| 0.316 | 0.3373 | 0.3146 | 0.3172 |
| 0.328 | 0.3488 | 0.317 | 0.3061 |
| 0.3343 | 0.3544 | 0.318 | 0.3004 |
| 0.341 | 0.3601 | 0.3189 | 0.295 |
| 0.3477 | 0.3655 | 0.3201 | 0.2898 |
| 0.362 | 0.3757 | 0.3218 | 0.2808 |
| 0.3922 | 0.3935 | 0.3158 | 0.3115 |
