# Supplementary material for: Chromatic Illumination Discrimination Ability Reveals that Human Colour Constancy Is Optimised for Blue Daylight Illuminations
Source: PLoS One. 2014 Feb 19;9(2):e87989. doi: 10.1371/journal.pone.0087989 (PMC3929610; doi:10.1371/journal.pone.0087989)
Supplement: Table S2 — CIE 1931 xy chromaticity coordinates of the fruit and matched printed papers used in the experiment, measurements taken under D67. (DOCX) [file pone.0087989.s005.docx]

Table S5. CIE 1931 xy chromaticity coordinates of the fruit and matched printed papers used in the experiment, measurements taken under D67.

|  | **Fruits** | | **Papers** | |  |
| --- | --- | --- | --- | --- | --- |
|  | **x** | **y** | **x** | **y** | **ΔE_uv_** |
| **Pear Reading 1** | 0.302 | 0.341 | 0.301 | 0.362 | 8.58 |
| **Pear Reading 2** | 0.352 | 0.43 | 0.315 | 0.406 | 13.98 |
| **Pear Reading 3** | 0.318 | 0.365 | 0.317 | 0.399 | 12.61 |
| **Pear Reading 4** | 0.304 | 0.34 | 0.301 | 0.362 | 9.42 |
| **Banana Reading 1** | 0.447 | 0.458 | 0.447 | 0.458 | 0.00 |
| **Banana Reading 2** | 0.434 | 0.457 | 0.434 | 0.457 | 0.00 |
| **Banana Reading 3** | 0.443 | 0.465 | 0.443 | 0.465 | 0.00 |
| **Apple Reading 1** | 0.503 | 0.348 | 0.499 | 0.353 | 4.28 |
| **Apple Reading 2** | 0.506 | 0.348 | 0.476 | 0.353 | 17.18 |
| **Apple Reading 3** | 0.439 | 0.352 | 0.465 | 0.355 | 12.13 |
| **Mean** |  |  |  |  | 7.82 |
